# Pregnancy-Related Precancerous Cervical Lesions: Pathogenesis, Diagnosis, Evolution, and Impact upon Gestation and Fertility

**DOI:** 10.3390/jcm13226718

**Published:** 2024-11-08

**Authors:** Teodora Ana Balan, Raluca Anca Balan, Demetra Socolov, Vlad Radu Gheorghiță, Tudor Andrei Buțureanu, Ioana Păvăleanu, Elena Teona Coșovanu, Irina-Draga Căruntu

**Affiliations:** 1 Department of Morphofunctional Sciences I, “Grigore T. Popa” University of Medicine and Pharmacy, 700115 Iasi, Romania; balan.teodora-ana@d.umfiasi.ro (T.A.B.); demetrasocolov@gmail.com (D.S.); tudorandreib@gmail.com (T.A.B.); ioana_pavaleanu@yahoo.com (I.P.); cosovanu_elena-teona@d.umfiasi.ro (E.T.C.); irinadragacaruntu@gmail.com (I.-D.C.); 2 “Elena Doamna” Clinical Hospital of Obstetrics and Gynecology, 700398 Iasi, Romania; 3 “Cuza Voda” Clinical Hospital of Obstetrics and Gynecology, 700038 Iasi, Romania; 4 L’HôpitalPrivé de l’Aube, 10000 Troyes, France; vladradu1@yahoo.com; 5 Department of Pathology, “Dr. C. I. Parhon” Clinical Hospital, 700503 Iasi, Romania

**Keywords:** cervical precancer, HSIL, HPV, pregnancy

## Abstract

More common than cervical cancer, cervical intraepithelial neoplasia (CIN) represents a precursor lesion of cervical carcinoma, being associated with HPV infection. Due to the bidirectional relationship between HPV and estrogen and progesterone in pregnancy, most of the published data claim that precancerous lesions remain stable or even regress during pregnancy, although several studies have indicated the tendency of HSILs to persist. It is considered that pregnancy-related cervical precancerous lesions undergo a postpartum regression, due to stimulatory effects of the immune microenvironment. Due to the rarity of publications on this subject, we aimed to offer a concise overview of and new insights into the current knowledge regarding the pathogenesis, diagnosis, and evolution of pregnancy-associated precancerous lesions, as well as their impact upon gestation and fertility.

## 1. Introduction

Cervical cancer ranks as the fourth most common cancer diagnosis globally, being one of the leading causes of death among women [1,2]. The second most frequently diagnosed malignancy in pregnancy after breast cancer, with an estimated incidence of 0.1 to 12 cases per 10,000 pregnancies [3,4,5,6], cervical cancer has seen a consistent rise in incidence over the last 20 years [7]. Cervical intraepithelial neoplasia (CIN), a precursor of cervical cancer, is ten times more common than cervical cancer itself, peaking in prevalence among women aged 20–34 [2,8]. This increase in incidence is likely due to the integration of cervical cancer screening into prenatal care and the coincidence of the peak incidence of cervical intraepithelial neoplasia with childbearing years [9].

CIN includes low-grade and high-grade intraepithelial lesions, primarily caused by high-risk human papillomavirus (HPV) types 16 and 18 [10]. HPV 16 and 18 can be traced in 25% of low-grade lesions, 50–60% of high-grade lesions, and 70% of cervical cancers [11]. Early detection through screening methods like Pap smears and HPV DNA testing is crucial for prevention. Abnormal findings in cervical cytology during pregnancy and the 1 year postpartum period occur at similar rates to the non-pregnant population: about 2% to 7% of pregnant women exhibit abnormal cytology, while the occurrence of high-grade lesions (CIN2+) during pregnancy is roughly estimated at 1% of expectant mothers [12,13].

Despite awareness campaigns, many women do not undergo routine gynecological exams, often having their first exam during pregnancy, which may lead to diagnosing more advanced lesions [14]. This aspect can also be caused by the hormonal changes of pregnancy, which can lead to immunosuppression, possibly increasing HPV infection risk [15]. Hormone-induced changes during pregnancy, such as the Arias–Stella phenomenon, as well as the specific increased vascularity and gland hypertrophy, can resemble high-grade squamous intraepithelial lesions (HSILs), making cervical screening challenging [4,14,16]. Additionally, pregnancy may complicate cervical smear analysis due to the presence of inflammatory and decidual cells [14]. These pregnancy-related changes can make abnormal cytology findings harder to evaluate or even lead to false-positive results if not recognized as physiological [4,16]. As an addition to this challenge, patients are usually asymptomatic, though some may present with irregular vaginal bleeding or contact bleeding [11].

The management of abnormal findings in pregnant women closely aligns with recommendations for non-pregnant women. However, endocervical curettage is not advised in any diagnostic circumstance. Routine cervical smears during pregnancy, especially for first-time testers, are recommended and even though pregnancy-related changes in the cervix can make Pap smear interpretation more challenging, this test remains a crucial and dependable diagnostic tool during pregnancy [17,18,19]. Moreover, repeating Pap smear screening postnatally is essential due to the common persistence of high-grade lesions [16].

Colposcopy plays a critical role in assessing CIN during pregnancy, but it can be challenging. The physiological enlargement of the cervix and the local edema during pregnancy makes specimen collection more difficult [4,15,20]. Inflammation, increased vascularization, and increased epithelial vulnerability may complicate the distinction from invasive processes, which may lead to an overestimation of pregnancy-related colposcopic findings [21,22,23]. Moreover, pregnant women often face challenges in cervical cancer screening due to concerns about miscarriage and premature delivery, especially among women with a history of vaginal bleeding during pregnancy [15]. Hence, it is imperative for colposcopy to be conducted by experienced clinicians [20].

Regarding the progression of cervical intraepithelial neoplasia during pregnancy, the literature suggests an inclination toward increased regression postpartum, likely linked to immune system restoration or stimulation. Some studies propose that vaginal delivery-induced microlesions and inflammation contribute to lesion regression, but these findings are debated [13].

The purpose of this review is to offer a concise overview of, and new insights into the current understanding regarding the pathogenesis, diagnosis, and evolution of pregnancy-associated precancerous lesions, thus exploring the impact upon gestation and fertility. This topic has limited publications due to its rarity. To conduct this review, a comprehensive literature search was performed across major scientific databases including Web of Science, Scopus, Science Direct, Google Scholar, the Cochrane Database for Systematic Reviews, and PubMed/Medline. The search utilized keywords such as ‘cervical precancer’, ‘HSIL’, ‘HPV’, and ‘pregnancy’ in various combinations. Subsequently, the relevant English-language literature spanning a 56-year timeframe was evaluated, with inclusion criteria focused solely on pregnancy-related cases of precancerous lesions.

## 2. Pathogenetic Mechanisms and Risk Factors

Sexual activity and HPV infection have been identified as the primary and most significant risk factors for cervical neoplasia. Research indicates that the risk escalates with a greater number of sexual partners, with studies revealing that at least 80% of sexually active women acquire genital HPV by age 50 [24]. The literature shows that HSILs exhibit similar epidemiological characteristics in both pregnant and non-pregnant women, inferring that the epidemiological factors associated with HSILs during pregnancy are similar to those observed in non-pregnant women [25].

Several risk factors contribute to the development of cervical cancer, including the age at first intercourse, the number of sexual partners, sexually transmitted infections, multiparity, and smoking [26]. Although smoking is considered to be the most significant environmental risk factor [27], a study conducted by Fader et al. demonstrated that smokers did not exhibit a higher likelihood of presenting high-grade lesions or undergoing a cervical procedure postpartum compared to nonsmokers; however, they were less prone to experience spontaneous regression of cervical dysplasia in comparison to nonsmokers [28]. For instance, Mukhtar et al. demonstrated that age and multiparity correlated with a higher risk of abnormal precancerous lesions [29]. In another study by Fan et al., logistic regression analysis revealed associations between abnormal Pap smears and the age of first intercourse, number of sexual partners, and smoking, the author concluding that the risk factors for abnormal Pap smears were consistent with those observed in non-pregnant women [30]. Conversely, Lertcharernrit et al. identified low BMI or multiple sexual partners as factors associated with abnormal Pap smears during pregnancy [31].

### 2.1. Pregnancy-Related Changes and Their Impact upon Cervical HPV Infection

It has been speculated that hormonal and immunological alterations during pregnancy could potentially facilitate the integration of HPV DNA and the progression of infection, as pregnancy causes physiological changes that result in temporary immunomodulatory effects, thus downregulating the expression of inflammatory chemokines [13,32,33]. In this way, several authors theorized that the increased rate of regression is attributed to the eradication of high-risk HPV (hrHPV) following vaginal delivery, as the cervical trauma experienced during the second and third stages of labor, as well as during delivery, might trigger an inflammatory response in the cervical epithelium, thereby promoting repair mechanisms. Another theory suggests that transient ischemic changes in cervical tissues during ripening could contribute to the regression of lesions [21,34]. In what concerns hormonal changes, one study suggests that sex hormones are overexpressed during pregnancy, potentially promoting cervical carcinogenesis by initiating squamous metaplasia in the transformation zone and modifying the local immune system. These hormone levels decrease after delivery, which could help explain the increased regression observed during that period [35].

At present, the precise impact of pregnancy on the course of HPV infection remains unclear. The “paraphysiological” immunological tolerance characteristic of pregnancy may theoretically facilitate infection or diminish the effectiveness of the immune system in clearing the infection. Generally, immunosuppressed individuals are at a higher risk for HPV infection, the onset of HPV-related lesions, and the development of cervical cancer [36,37]. Conversely, an active immune response in the host is associated with virus elimination and lesion regression, even in individuals undergoing immunosuppressive treatment [38,39,40]. In accordance with these immunological factors, some studies have observed a higher incidence of high-risk HPV subtypes in pregnant women compared to non-pregnant women. Additionally, it has been noted that younger mothers and women with higher parity have elevated rates of HPV infections, while age over 25 years, HSILs, and HPV type-16 infections are significant risk factors for the persistence or progression of cervical intraepithelial lesions in the postpartum period [21].

#### 2.1.1. Pregnancy Hormonal Particularities

**Figure 1:**
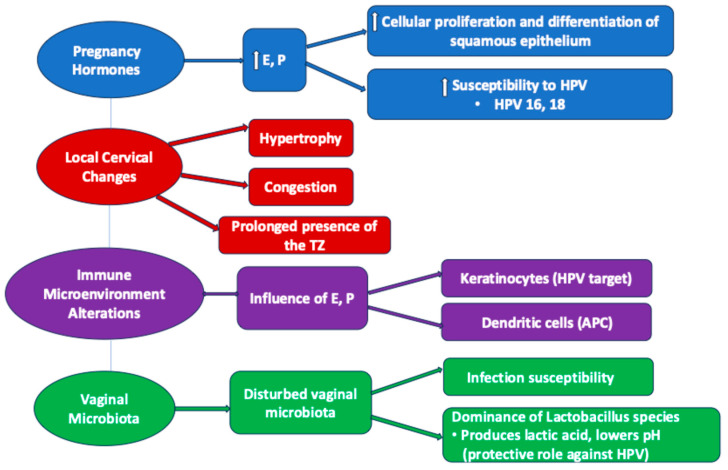
Hormonal and immunological changes in pregnancy affecting HPV infection. E—estrogen; P—progesterone; HPV—human papilloma virus; TZ—transformation zone; APC—antigen-presenting cell.

Pregnancy induces significant hormonal changes, particularly elevated levels of estrogen and progesterone. These hormones influence the cervical epithelium by promoting cellular proliferation and differentiation, potentially increasing susceptibility to HPV infection and the development of precancerous lesions, accelerating the evolution of high-risk HPV cervical infection, especially type 16 [41]. In this regard, estrogen stimulates the proliferation of the squamous epithelium, while progesterone supports its differentiation. During pregnancy, elevated levels of these hormones enhance the regenerative capacity of the cervical epithelium, creating a milieu conducive to the persistence and integration of oncogenic HPV types, particularly HPV 16 and HPV 18 [42]. The transformation zone’s heightened activity may increase the likelihood of dysplastic changes in response to HPV-induced cellular disruption (Figure 1). The assumed hormonal mechanisms are correlated and responsible for several gravidity-related local changes.

##### Disturbed Vaginal Microbiota

During pregnancy, an imbalance of the vaginal flora occurs, that together with the humidified environment of the specific area favors the occurrence of infections with various agents, including HPV.

The vaginal microbiota is strongly influenced by gestation, sometimes leading to pregnancy complications. The abundant vaginal microbiota decrease during gestation, being dominated by *Lactobacillus* species with advancing pregnancy. A large longitudinal study showed that besides the main factors which influence the vaginal microbiota in pregnancy, represented by gestational age and individual identity, there are other different factors which can alter the composition of vaginal flora such as parity, maternal age, obesity, or cannabis use [43]. Moreover, the local immunoproteome (chemokines, cytokines, defensins) interacts with the vaginal ecosystem, the immune response being at least as varied as the vaginal microbiota [43].

It has been underlined by numerous studies that the vaginal microbiota play a role in the persistence of HPV in the human vagina, as well as in the development and progression of cervical intraepithelial neoplasia (CIN). Another well-known aspect is that *Lactobacilli* confer a protective role by lowering the vaginal pH due to the production of lactic acid (Figure 1). As Lactobacillus species dominate the vaginal microbiota across the pregnancy, it can be stated that on the one hand, pregnancy offers a protective role against viral infections, including HPV; on the other hand, there may be an opening to therapeutic opportunities with new agents like probiotics to prevent HPV infection and the risk of developing cervical dysplastic lesions [44].

##### Anatomical Modifications of the Cervix

During pregnancy, the cervix increases in size due to hypertrophy and congestion (Figure 1). Consequently, the squamous–columnar junction undergoes changes, so that the transformation zone will persist for a long time at the level of the exocervix, which will cause an increased susceptibility of the metaplastic stratified squamous epithelium to HPV infection and the development of precancerous lesions [41].

Moreover, it is known that in humans and mice, the endocervix possesses both squamous and columnar epithelial cells, which differ through the keratin markers Krt5 and Krt8, respectively [45].This was demonstrated by a study which revealed that non-pregnant and pregnant mice contain cervical secretory epithelial cells which express the columnar cell type Krt8 and the squamous marker Krt5 [46]. These aspects can translate a plasticity of squamous cells that can differentiate into the phenotype of Krt8+ secretory cells. Future studies are needed to elucidate the various existing hypotheses, which refer either to the transformation of squamous progenitor cells into secretory columnar cells, or to the birth of the different epithelial cell subtypes encountered in pregnancy from multiple progenitor cells, or even the birth of squamous and columnar cells from the same cervical bipotent stem cell [46].

#### 2.1.2. Cervical Immune Microenvironment Alterations

During pregnancy, estrogens and progesterone also influence the immune microenvironment at the level of the cervix, increasing the susceptibility of the transformation area to malignancy. The cervical squamous epithelium contains keratinocytes, which are the main target of HPV, and immature Langerhans dendritic cells, which are involved in immune surveillance of the epithelium [41]. Thus, estrogens and progesterone influence the role of the APC (antigen-presenting cell) of these cells, possibly due to the production of TGF-β which does not allow the maturation of dendritic cells, or due to the decrease in the recruitment of dendritic cells [41] (Figure 1). In addition, at the transformation zone the conversion of estradiol into 16a-hydroxyestrone [47] takes place, which binds covalently and activates ERα (estrogen receptor alpha), necessary in carcinogenesis. Once activated, ERα binds to the responsive elements of the LCR (long control region), inducing the transcription of E6 and E7 in order to support HPV gene activity [48]. It can thus be deduced that there is a stimulating interrelationship between HPV and estradiol, considered as the strongest factor in this type of carcinogenesis, either directly through the estrogen responsive elements of the viral genome or through an uncontrolled cell proliferation that will stimulate malignant proliferation [41,45].

Because of its mitogenic activity, estrogen stimulates the proliferation of keratinocytes by increasing cyclin D2 and inducing the progression of the G1 phase to the S phase of the cell cycle [49]. On the other hand, estrogen inhibits apoptosis induced by oxidative stress in keratinocytes by stimulating the expression of the anti-apoptotic protein bcl-2 [50]. Another known role of estrogen is the involvement in the production of cytokines [41]. Thus, estradiol inhibits the expression of GM-CSF in the U2OS cell line due to the interaction with ERα, decreasing this production through contact with ERβ [51].

Progesterone also influences cytokine production, affecting the immune system [41]. The increase in progesterone during pregnancy causes immune suppression, thus increasing the predisposition to the development of HPV-associated precancerous lesions [41]. Progesterone increases the production of IkBα and inhibits GM-CSF secretion [41]. High levels of progesterone during pregnancy enhance HPV gene expression, increasing the number of viral copies and multiplying virally transformed cells [41]. Therefore, in the first trimester of pregnancy there is a higher frequency of viral persistence due to a low immune response to HPV, a situation that knows a serious recovery at the beginning of the third trimester, with a stimulation in the postpartum period that will lead to the regression of the infection [41].

It has been demonstrated that estrogens and progesterone increase the level of pro-apoptotic HPV E2 and E7 proteins. As the E2 gene is lost in cervical carcinogenesis due to the integration of HPV DNA in the host genome, estrogen and progesterone represent a possible risk factor for malignant transformation in the uterine cervix [41].

## 3. Screening Strategies During Pregnancy

Cervical cytology screening programs, already well-implemented in developed countries, can significantly reduce the occurrence of cervical cancer. Anomalies detected in cervical cytology prompt primary care physicians to refer patients to specialists in colposcopy for the assessment of precancerous lesions identified as cervical intraepithelial neoplasia [11].

Most women from low/low-middle income countries do not undergo screening during pregnancy due to a lack of clear information, coupled with concerns about bleeding or miscarriage. Visualization of the cervix can be sometimes difficult, with challenges increasing as the pregnancy progresses. Excessive discharge rarely poses problems, while pain and discomfort are less common in earlier gestational ages [52].

Among the sampling tools, the cyto-broom is preferred, whereas the endocervical brush is often avoided due to its associated risk of bleeding, which may cause distress, even though the Pap smear performance is better when using it [52].

The early second trimester is considered an optimal time for screening, as endocervical cells are translocated outside the cervix, making the transformation zone easier to visualize and sample. Studies have shown that the endocervical sampler used in conventional Pap smears, which only reaches the lower half of the cervix, is safe during pregnancy [12,14].

Routine antenatal and postpartum care should involve reviewing a woman’s cervical screening history, with screening provided for those who are due or overdue. Screening is safe to conduct at any stage of pregnancy as long as the appropriate sampling tools are used. According to American and Australian guidelines, pregnant women who test positive for high-risk HPV (types 16/18) should be referred for early colposcopy. For other cases, cytology triage can be conducted, and if high-grade squamous intraepithelial lesions (HSILs) or glandular abnormalities are detected, early colposcopy should be advised. Women with a colposcopic diagnosis of CIN2/3 can either be reassessed postpartum or undergo surveillance colposcopy every 12–24 weeks [19,23].

The most recent Canadian guidelines recommend that pregnant women who are high-risk HPV-positive with normal or low-grade cytology (ASCUS or LSILs) should have HPV-based screening repeated 3 months after delivery. Those with high-risk HPV and reflex high-grade or glandular cytology (ASC-H, AGC, or HSILs) should be referred to colposcopy within 4 weeks [20].

French guidelines claim that the frequency and the test used for cervical screening are the same for pregnant and non-pregnant women, according to their age [52,53].

In pregnancy, it is recommended that the detection test be carried out only at the first pregnancy control in the first trimester, as testing in the second or third trimester is not recommended. If the screening test was not performed in the first trimester of pregnancy, it is recommended that it be postponed for the postpartum period [53].

Following an abnormal cytological test, the recommended strategy is carried out depending on the woman’s age, as follows: (i) for women under 30 years old, for the diagnosis of LSILs or ASCUS, a new cytological test is recommended 2–3 months after birth, and for the diagnosis of ASC-H, AGC, or HSILs, it is recommended to perform a colposcopy immediately after the diagnosis; (ii) for women over 30, a positive HPV test accompanied by an abnormal cytology does not yet benefit from specific good clinical practice recommendations [54].

## 4. Diagnostic Particularities of HSILs in Pregnancy

Pregnancy involves a special population in which the diagnostic and therapeutic approach must take into account, on the one hand, the risk to the mother and fetus, and on the other hand, the risk of omitting a cancer diagnosis. It is considered that cancer progression rate is not different in pregnancy [55].

Cervical intraepithelial neoplasia can manifest at any stage and may progress or regress over time. It has been previously categorized into three distinct entities, with grade 1 cervical intraepithelial neoplasia (CIN1) signifying mild dysplasia or abnormal cell growth, typically confined to the lower third of the epithelium; grade 2 (CIN2) reflecting moderate dysplasia restricted to the lower two thirds of the epithelium; and grade 3 (CIN3) indicating severe dysplasia, affecting more than two thirds of the epithelium [56].

The 2020 WHO classification of female genital tumors describes three distinct entities of cervical intraepithelial neoplasia. The essential diagnostic criteria for LSILs (CIN1/condyloma) describe full epithelial thickness atypia with moderate to abundant cytoplasm in the cells located in the upper two thirds of the epithelium, as well as basaloid morphology alongside significant mitotic activity confined to the lower third of the epithelium; as for the highly desirable diagnostic criteria, koilocytic atypia should be present in the middle and surface cells. For HSILs (CIN2), the essential diagnostic criteria consist of full epithelial thickness atypia, with basaloid morphology and mitotic activity extending into the upper half or upper two thirds of the cervical epithelium, koilocytic change being retained on the surface. As for HSILs (CIN3), the authors describe full epithelial thickness atypia, the base of the lesion being frequently difficult to distinguish from the surface, with mitotic activity identifiable throughout the entire epithelial thickness and a significantly higher nuclear–cytoplasmic ratio than in CIN1 and CIN2 in the upper layers of the epithelium [57].

High-grade squamous intraepithelial lesions are typically identified in patients aged between 25 and 35 years, while invasive cancer tends to develop 8 to 13 years after the initial diagnosis of these lesions [11].

Patients typically do not show symptoms, although some may experience irregular or postcoital bleeding. Abnormal cervical cytology is commonly detected through Papanicolaou smears [11]. In general, both conventional and liquid-based cytological Papanicolaou tests have shown comparable diagnostic accuracy in both pregnant and non-pregnant individuals, with a sensitivity ranging from 70% to 80% for detecting HSILs [58,59].

In terms of HPV testing, Kim et al., authors of a Korean study involving 15,141 women who underwent both HPV testing and cervical cytology, concluded that HPV testing exhibited greater sensitivity than cytology. However, the specificity of HPV testing varied depending on the methods used [60].

While HPV DNA testing is typically conducted when atypical squamous cells of undetermined significance (ASC-US) are present, certain programs perform high-risk HPV DNA testing alongside Pap smears in all women aged over 30 (co-testing screening) [61,62,63]. Consequently, a woman may receive a normal Pap test result but test positive for high-risk HPV. Studies show that in such cases, the likelihood of having CIN2 or higher lesions is 4%, and these women often experience only transient HPV infections [64]. The American Society of Colposcopy and Cervical Pathology (ASCCP) advises non-pregnant women with positive high-risk HPV and negative Pap smear results to repeat co-testing after 12 months; for pregnant women, it is advisable to have a repeat co-testing at the 6-week postpartum visit [21,65,66].

Moreover, Gill et al. underscored the significance of conducting Pap smear tests during the postnatal period, as it became evident that screening results obtained during pregnancy could yield false positives. Hence, it is crucial to repeat Pap smear tests during the postnatal period before considering any treatment interventions [16]. Traditionally, postpartum visits for cervical cancer screening have been done 6 to 8 weeks after childbirth, some studies showing comparable outcomes when screening is performed 2 to 3 weeks postpartum [67].

Management guidelines for pregnancy-related cervical dysplasia are not very clearly established, as they are generally based on data from non-pregnant women or results of retrospective studies on pregnant women [68].

Currently, the use of markers such as Ki67, p16, CK 13/14, or p53 is widely known to assess the regressive or progressive character of a high-grade squamous intraepithelial lesion (CIN2, CIN3) [68], with dual p16/Ki67 immunostaining representing an important test in the diagnostic evaluation of a persistent HPV lesion.

One study revealed that dual immunostaining p16/Ki67 in pregnant women with CIN2/3 is more variable than in non-pregnant women [68].

These results can be correlated with the specific gestational status that can interfere with the expression of cell cycle proteins and the process of carcinogenesis. The modulation of the expression of the two markers is due to the increase in the level of progesterone, which influences the gene expression of proteases, cell adhesion molecules, transcription factors, or inflammation regulators. In this sense, it can be stated that cervical dysplasia in pregnant women can have a less aggressive behavior compared to similar lesions in non-pregnant women [68]. Moreover, in order to accurately interpret the variability of the variable dual expression of p16 and Ki67 in pregnant women, it is necessary to create an algorithm that takes these correlations into account [68].

Cervical cancer and CIN occur in pregnant women at similar rates as in non-pregnant women, with around 14% of cervical abnormalities during pregnancy being categorized as HSILs. Pregnancy itself does not increase the risk of developing CIN, and the likelihood of CIN progressing to invasive cancer during gestation is low [25].

When dealing with abnormal Pap smears, the preferred approach is colposcopy accompanied by targeted biopsy and histological assessment of cervical abnormalities suggestive of dysplasia. If biopsy confirms CIN2/3 or if the colposcopist is confident of the absence of invasion based on colposcopic assessment alone, regular monitoring through repeat cytology and colposcopy every 12 weeks is advised in order to track disease progression [4]. In addition, high-risk HPV-positive women should have a colposcopy 6 weeks postpartum, whereas those who test negative for high-risk HPV can be monitored with cytology at the same postpartum interval [21].

The criteria for undergoing colposcopic examination are comparable for both pregnant and non-pregnant patients [21]. Pregnant women showing clinical and cytological signs suggestive of cervical cancer(except for low-grade squamous intraepithelial lesions) should undergo colposcopy, with or without biopsy, as they can be performed safely during pregnancy. However, if feasible, biopsy should be avoided due to the potential rare complications, endocervical curettage being strongly contraindicated [4,7]. While there is no evidence suggesting that performing a cervical biopsy on a pregnant patient carries a higher risk compared to a non-pregnant patient [18,69], concerns over potential bleeding from the hyperemic and congested pregnant cervix or the initiation of preterm childbirth often deter physicians from conducting biopsies in such cases [7,19,70]. Thus, biopsies are typically reserved for situations where the results could significantly impact the patient’s management, such as lesions suspicious for invasive cancer as observed during colposcopy [65]. Some experts propose performing biopsies during the second trimester to minimize any potential association with spontaneous miscarriage, while others suggest using a stiff brush as a less invasive alternative for diagnosis [71]. The management of excessive bleeding post-biopsy includes applying prolonged pressure with a large swab and, if necessary, using a hemostatic agent like Monsel’s paste or silver nitrate, with the mention of utilizing the smallest effective quantity due to their caustic nature. In cases of excessive or uncontrollable bleeding, electrosurgical cauterization, fine suture, or vaginal packing can be considered. Despite these potential complications, adverse effects still remain uncommon, with the literature data showing that pregnancy does not represent a major impediment for performing cervical biopsy [25,72].

It is crucial to recognize that in pregnant women, the morphological changes in the cervix leading to diagnostic challenges and the potential risks associated with diagnostic and therapeutic procedures, both maternal and fetal, require a thorough management by competent physicians [7,21], being mandatory for the gynecologist to provide all available and eloquent clinical data to the pathologist. Physiological changes in the cervix during pregnancy can promote changes in squamous and glandular epithelial cells. Thus, pregnancy-related hormonal changes including inflammation, hypertrophy, microglandular hyperplasia of the endocervical glands, degenerated decidual cells, atypical endometrial glandular cells with hypersecretory appearance (Arias–Stella reaction), trophoblastic cells displaying cytoplasm with varying staining patterns, enlarged nuclei, or even immature metaplastic cells may resemble HSILs, potentially leading to false-positive results in case the pathologist is uninformed about the patient’s pregnancy status [4,7,21,29].

Moreover, the ASC-H category is related to cytological aspects of immature (atypical) squamous metaplasia, which is challenging to interpret. Pregnancy contributes even more to the diagnosis of an ASC-H type lesion, due to the specific anatomical and physiological changes during this period, which involves a more accentuated squamous metaplasia process, leading to a smear richer in metaplastic cells, reactive changes, and inflammation. However, the management guideline is not different in pregnant women or in the postpartum period compared to non-pregnant women, although colposcopy is more difficult to perform during pregnancy [73].

In this regard, one study revealed that the ASC-H category during pregnancy is difficult to assess, which is why in this situation a conservative management with adjunctive HPV testing is considered a proper approach [73].

The colposcopic presentation of the cervix undergoes significant changes during pregnancy, becoming easier to evaluate due to the improved visibility of the squamous–columnar junction (SCJ) and the transformation zone (TZ) resulting from the physiological eversion of the columnar pattern. However, edema, cyanosis, friability, increased pelvic congestion of the cervix, and protrusion of the vaginal walls can impose objective constraints on the subjective interpretation of colposcopy. Additionally, abundant thick mucus production is commonly observed, obstructing the complete visualization of the cervix around the external os in some cases. To optimize visualization, it is crucial to use the largest speculum possible tolerated by the patient. Cervical mucus is typically thick, opaque, and tenacious, and twisting it around a dry cotton swab is sometimes helpful. While these changes may significantly alter colposcopic findings, some authors argue that there is minimal disparity in the colposcopic appearance of cervical dysplasia between pregnant and non-pregnant women [21]. Conversely, others suggest that pregnancy-induced alterations can exaggerate the severity of colposcopic lesions, the literature showing that white epithelium appears more commonly in pregnant women diagnosed with HSILs [23,25,69]. As pregnancy progresses, stromal decidualization becomes more pronounced, manifesting colposcopically as densely aceto-whitening plaque-like lesions with spider-like superficial blood vessels, frequently forming a ring-shaped aceto-whitening decidualized area surrounding normal capillaries, resulting in a “starry sky” appearance. Additionally, active immature metaplasia can produce thin aceto-whitening areas with fine mosaic and punctuation vessels, making them challenging for unexperienced colposcopists to distinguish from low-grade intraepithelial lesions (LSILs) [21]. Moreover, the increased cervical vascularity during pregnancy may enhance the acetic acid reaction, likely leading to minor alterations being mistaken for major ones even by experienced colposcopists [25].

In this regard, considering cervical hyperemia and other local physiological changes specific to pregnancy, the examination of pregnant women should be performed by an experienced colposcopist who can differentiate the lesional aspects from those related to pregnancy [55].

If the initial colposcopic assessment is deemed inadequate during the early stages of pregnancy, it is advisable to repeat it in the second trimester when there is a higher probability of detecting complete eversion of the SCJ and TZ [23]. To enhance visualization, ring forceps can be utilized as an alternative to an endocervical speculum to dilate the external os and facilitate thorough examination of the endocervical canal. However, if the TZ remains incompletely visualized, the decision to proceed with a diagnostic conization should carefully weigh the potential risks of complications against the possibility of an underlying malignancy [73].

According to the current consensus guidelines of the American Society of Colposcopy and Cervical Pathology (ASCCP), colposcopy is postponed until 6 weeks after childbirth in patients with LSIL cytology. This delay is recommended because these abnormalities typically regress spontaneously and are unlikely to conceal a hidden invasive malignancy [21]. The ASCCP guidelines advocate for an overall conservative approach towards abnormal Pap smears in the absence of clear evidence of invasive cancer during pregnancy [74]. A conservative management approach for HSILs during pregnancy involves colposcopic evaluation both during gestation and postpartum, irrespective of the mode of delivery, as colposcopy is reasonable for patients experiencing persistent dysplasia or for those who might encounter challenges in accessing healthcare facilities after childbirth [21,25]. In what concerns ASC-US (atypical squamous cells of undetermined significance), ASC-H (atypical squamous cells that cannot exclude a high-grade lesion), as well as AGC (atypical glandular cells) and AIS (adenocarcinoma in situ), colposcopy is the preferred method of evaluation as well, playing a crucial role in guiding cervical biopsies of suspicious lesions [21]. Typically, if invasive cancer can be ruled out, all diagnostic and therapeutic interventions are postponed until after delivery [74].

The ASCCP 2019 consensus guidelines recommend colposcopy in pregnant women with a high risk of CIN3+ lesions due to the lack of previous screening or a persistent HPV infection. At the same time, the colposcopic examination is postponed for pregnant women with a previous colposcopy without a diagnosis of CIN2+ or for whom the previous HPV test is negative [55].

In this way, treatment procedures like ablation or excision are not recommended during pregnancy. However, studies show that the loop electrosurgical excision procedure (LEEP) is safe in the first 15 gestational weeks, demonstrating that the risk of miscarriage and severe bleeding is minimal [75]. Despite the elevated risk of complications associated with diagnostic excisional procedures such as conization, they are considered when uncertainty persists, such as high suspicion for microinvasive disease, unsatisfactory Pap smear or colposcopy, or discordance between cytology and colposcopy findings. The potential obstetric complications, including hemorrhage, cervical incompetence, and fetal loss, should be discussed with the patient before proceeding with any intervention [4,25].

ASCCP 2019 consensus guidelines recommend that during pregnancy, the management of cervical abnormal results should be similar to that of non-pregnant women, following identical clinical thresholds for colposcopy and surveillance. Thus, endometrial and endocervical biopsy and curettage, as well as treatment without biopsy are not acceptable in pregnancy. If cancer is suspected after the cytological, colposcopic, or histological examination, an excisional diagnostic procedure or a new biopsy is recommended. If the first colposcopy during pregnancy detects an HSIL (CIN2 or CIN3), the patient will be followed-up colposcopically and tested (HPV or cytology, depending on age), every 12–24 weeks. Postponing colposcopy for the postpartum period is also accepted. If an invasive lesion is suspected or the existing lesion worsens, a new biopsy is recommended. Treatment of histological HSILs(CIN2 or CIN3) is not recommended. In the case of the diagnosis of an adenocarcinoma in situ (AIS) during pregnancy, it is accepted that either the patient is directed to a gynecological oncologist, or that the therapeutic management is carried out by an experienced gynecologist [55].

Postpartum colposcopy is advisable, but at least 4 weeks after birth. If the colposcopic result is positive in patients diagnosed with HSILs during pregnancy (CIN2 or CIN3), a full diagnostic evaluation (HPV testing, cervical cytology, and biopsy) or an excisional therapeutic procedure is accepted. If the colposcopic result is negative, a precipitated therapeutic approach without a complete diagnostic evaluation is not recommended [55].

## 5. The Bidirectional Relationship Between Cervical Intraepithelial Lesions and Pregnancy

### 5.1. The Effect of Pregnancy on High-Grade Cervical Intraepithelial Lesions

The course of HSILs during pregnancy remains a topic of debate in the literature. Reported rates of persistence, regression, and progression vary considerably across different studies. Most of the available literature consists of retrospective studies, with prospective trials being extremely rare [27].

While the incidence of gynecological precancerous lesions remains relatively low, the potential for progression to malignancy cannot be ignored. Kaplan et al. showed that most cases of LSILs either regress or remain stable during pregnancy, but several studies have indicated a notable likelihood of HSILs persisting, underscoring the necessity for ongoing colposcopic and cytological monitoring during pregnancy (around 20–30 weeks) and postpartum (after 6 weeks) [11,76]. Moreover, HPV is a primary factor contributing to the rise in precancerous lesions among young women. The usage of HPV DNA testing alongside colposcopy should also allow physicians to identify these lesions before progression [11].

Several studies have shown that pregnancy typically has minimal impact on cervical lesions and the likelihood of progression to invasive disease during pregnancy is extremely rare (0–0.4%), with most cervical intraepithelial lesions remaining stable or regressing [77]. Moreover, studies have indicated higher postpartum regression rates compared to spontaneous regression rates in non-pregnant women [78,79,80,81].

For cases of ASC-US or LSIL during pregnancy, the rate of detecting CIN2/3 during postpartum follow-up is merely 3.7% [82]. It is common for CIN2/3 lesions to spontaneously regress postpartum, with 48–70% experiencing regression during pregnancy [77,83,84]. Gomez et al. observed a regression rate of high-grade lesions at 43% with no cases of invasive lesions during postpartum colposcopy follow-up, aligning with rates documented in the previous literature [13,26,84]. Fader et al. noted an 86% regression of LSILs postpartum, with no invasive cancer cases identified [28]. Similarly, Kaplan et al. found that among patients with antepartum LSIL cytology, 62% experienced disease regression after delivery, 32% had persistent LSILs, while only 6% progressed to HSIL; no invasive cancer progression occurred during pregnancy [76]. On the other hand, Coppolillo et al. showed that 28 women with HSILs on first-trimester antepartum cytology underwent colposcopy and biopsy 6 to 8 weeks postpartum, with HSILs persisting in 89% of women and microinvasive disease being found in 11% of patients [85].

The literature showed variable data regarding the percentage of women with benign inflammatory changes (21–95%) [16,86]. Thus, in the study conducted by Gill et al., two out of the seven women with abnormal cervical cytology presented inflammatory changes during the postnatal period in spite of the regression of abnormal cytology [16]. Studies emphasized the importance of providing appropriate treatment for women with persistent inflammation to reduce the risk of developing cervical intraepithelial lesions, noting that due to the low sensitivity of Pap smears, premalignant lesions of the cervix might go undetected in women with inflammatory Pap smears [87,88].

Regarding the mode of delivery, there is limited and contradictory evidence regarding the impact of the delivery mode on regression rates of cervical dysplasia detected antepartum. While some studies suggest that the mode of delivery does not influence the regression of HSILs, several authors contradict this theory by publishing results that indicate that women who deliver vaginally show higher postpartum regression rates.

Candido Murta et al., in accordance with Schuster at al., indicated that the mode of delivery did not impact the prognosis of patients diagnosed with HSILs who underwent cytological and colposcopic evaluation antepartum and postpartum [25,89]. Throughout a follow-up period averaging 4.4 ± 4.3 years, no cases of invasive disease were found among the patients after treatment. Even though it has been suggested that the spontaneous regression of HSILs may occur due to trauma during delivery or alterations in maternal immune status following pregnancy, the study contradicts the notion of a high regression rate postpartum, as it observed that only 24% and 21.4% of patients did not exhibit HSILs during postpartum evaluation following vaginal delivery and cesarean section, respectively [25].

Yost et al. examined the histological regression and progression rates of CIN2 and CIN3 after delivery in both women who had vaginal deliveries and women who underwent cesarean section, finding similarly high postpartum regression rates (around 70%) regardless of delivery method [84].

Coppola et al. reported an 88% incidence of persistent disease, finding no correlation between delivery method and disease regression, and thus also indicating that the delivery method did not affect the regression rate and suggesting that a low regression rate is not solely attributable to vaginal delivery-induced trauma. Nonetheless, these findings underscore the importance of conducting additional cytological and colposcopic evaluations after delivery [90].

On the contrary, some studies indicate that the postpartum regression rates were influenced by the mode of delivery, showing a higher rate of spontaneous regression of abnormal cervical cytology detected antepartum for women who delivered vaginally.

In this way, Stuebs et al. reported a persistence rate of 69.9% for vaginal delivery and a 78.3% for cesarean section [27], while Chung et al. reported higher postpartum regression rates, more specifically 92.9% for women who delivered vaginally and 63.2% for women who underwent cesarean section, as well as a higher rate of progression for women with cesarean section (6.5% vs. 2.7%) [9]. Similarly, Siristatidis et al. presented findings consistent with this theory, comparing regression rates among patients with HSILs delivering vaginally and by cesarean section, reporting regression rates of 67% and 13%, respectively [91]. Likewise, Ahdoot et al. observed a higher regression rate of cervical dysplasia associated with vaginal delivery compared to cesarean section, although their analysis was based solely on cytological data [83].

The precise mechanisms behind the high rates of postpartum regression remain incompletely elucidated, the literature suggesting various theories, such as the potential induction of viral activation in women already infected with HPV, triggered by hormonal fluctuations during pregnancy, resulting in cervical dysplasia that subsequently regresses after childbirth. Furthermore, it is suggested that an immune system compromised during pregnancy may be reactivated postpartum [2].Moreover, the diet and nutritional intake represent modifiable factors affecting the risk of various cancers, and several variables, including calcium, zinc, iron, selenium, carotenoids, and vitamins A, B12, C, D, E, and K, have been shown to impact the regression rate of CIN. Various oligo-elements and micronutrients have displayed potential protective effects against cervical cancer by influencing different stages of the natural progression of CIN [92].

Additionally, an interesting finding was that patients who underwent emergency C-sections after entering the active phase of labor, which frequently involves cervical desquamation, showed evidence of cytological regression. This finding aligns with the concept that inflammatory reactions resulting from cervical trauma due to mechanical dilatation during the active phase of labor may contribute to the higher regression rates observed in patients who undergo vaginal delivery [9]. Proposed theories to explain this regression phenomenon include epithelial desquamation of the cervix or an enhanced localized immunological response [93]. In the research conducted by Fukuda et al., the authors noted impaired immune responses observed in cervical biopsy samples from women with persistent cervical dysplasia [52]. Specifically, they observed significantly lower counts of Langerhans and T helper cells in the biopsy specimens of women with persistent dysplasia, these cells being essential to the inflammatory wound healing process [52,79].

### 5.2. Impact of High-Grade Cervical Intraepithelial Lesions on Gestation and Fertility: Therapeutic Aspects

Treatment for cervical intraepithelial neoplasia can impact fertility in various ways. For instance, cervical canal stenosis can form as a physical barrier, hindering sperm from reaching the endometrial cavity and fallopian tubes. This complication is observed in approximately 8% of patients undergoing excisional treatment, compared to 1% undergoing ablative treatment. Additionally, the absence of cervical mucus may prevent sperm transportation from the upper vagina to the endometrial cavity. Depending on the extent of conization, CIN therapy may lead to infertility due to cervical incompetence and early pregnancy loss. Furthermore, tubal obstructions or dysfunction, leading to pelvic inflammatory disease, may arise due to ascending infections during the treatment process [11].

Moreover, in a study involving more than 435,000 women followed for up to 12 years, Naleway et al. found no negative impact of cervical treatment procedures on future pregnancy rates. Surprisingly, the authors noticed higher pregnancy rates among women who underwent these therapeutical procedures compared to those who did not, as well as compared to women who only underwent diagnostic procedures [93]. These results align with findings from another study, which also noted an elevated likelihood of pregnancy in the treatment group compared to a matched group of unexposed women [94].

In terms of gestational outcomes, an analysis of 2480 cases over an 11-year period found notable associations between the detection of high-risk human papillomavirus (HPV DNA) and instances of preterm births and placental abnormalities [95]. These findings suggest that detecting high-risk HPV cervical infections during pregnancy could serve as a predictive factor for adverse pregnancy outcomes.

In a population-based study by Loopik et al. covering 45,259 pregnancy outcomes, it was noted that both untreated and treated cervical intraepithelial neoplasia are associated with increased chances of spontaneous preterm birth compared to women without a CIN diagnosis. Additionally, the authors found that an excised total volume of less than 0.5 cc of cervical tissue did not show an association with preterm birth. However, logically, a higher volume of excised tissue was linked to a more than twofold increase in the likelihood of preterm birth, irrespective of the severity of the underlying cervical condition or other potential risk factors. Their findings indicated that in women with treated CIN, the risk of preterm birth before 37 weeks doubles compared to healthy controls [96].

Regarding local or systemic treatments, to the best of our knowledge, there are no specific scientific data or guidelines to support their use in pregnancy in the context of a diagnosis of CIN or a persistent HPV infection. However, Isoprinosine is not recommended during pregnancy or postpartum, due to the fact that it is excreted in milk, and its administration should carefully weigh the risks and benefits.

Over time, it has become increasingly evident that not only women who undergo excisional treatment for cervical intraepithelial neoplasia but also those managed conservatively for CIN may face a heightened risk of adverse perinatal outcomes. As the literature data show an increased risk for preterm birth in women with treated CIN compared to those with untreated CIN, such findings prompt consideration of whether cervical disease itself or underlying common risk factors contribute to preterm birth [96]. While some studies have shown no association between treated CIN and preterm birth [97,98,99,100], Loopik et al. suggested that the potential link to preterm birth primarily stems from the excisional procedure itself (biopsy or treatment) rather than the underlying cervical disease, as surgical intervention on the cervix can trigger preterm contractions leading to premature delivery [96].

In this way, Wiik et al. showed that women who previously underwent excisional treatment for CIN showed an elevated risk of preterm delivery (PTD), spontaneous preterm delivery, preterm premature rupture of membranes (pPROM), and term premature rupture of membranes (PROM) compared to women with a history of normal cytology and those with untreated CIN during pregnancy. Treated women with cone lengths up to 10 mm had a roughly 50% higher risk of PTD compared to those with normal cervical cytology or untreated CIN during pregnancy. The risk of PTD appeared to be rising by 15% with each additional millimeter of cone length. Another noteworthy finding was the correlation between increasing cone length and elevated risks of PROM at term, neonatal sepsis, and chorioamnionitis [101].

When considering treatment for women of reproductive age, clinicians aim to minimize excision size and avoid unnecessary diathermy after excision, in order to preserve the cervical tissue. Nonetheless, as treatment must effectively remove the CIN to prevent cancer development, this evolution may potentially have come at the expense of a less pronounced reduction in cervical cancer development among treated women [102]. This aspect underscores the importance of expertise in colposcopy, patient selection for treatment, and excisional treatment.

On another hand, only five studies provided outcomes on low birth weight (<1000 to 2000 g) [103,104,105,106,107]. A retrospective cohort study indicated an elevated risk for low birth weight and small for gestational age in women treated for CIN3 compared to those without CIN [108]. Loopik et al. indicated that women with treated CIN face more than double the odds for low birth weight (<1000, <1500, <2000, and <2500 g) compared to women without CIN [96]. Only two studies assessed the outcome of an APGAR score <7 at 5 min and found no statistically significant relationship, whereas our study revealed a 1.5 times statistically significant increased likelihood in women with treated CIN [109,110]. In what concerns perinatal death, a meta-analysis indicated a 1.5 times increased risk for treated women versus untreated women [111].

It is well established that HPV infection can be vertically transmitted, through prenatal transmission, from placenta or amniotic fluid, or perinatal transmission, through ascending infection during birth canal passage or after membranes rupture [112].

In this regard, one study revealed that birth canal passage represents the main mechanism for vertical HPV transmission, with only a few newborns through caesarean section being HPV positive [112]. Regarding the negative impact of HPV infection on the infant’s health, besides the well-known neonatal infections like respiratory and juvenile-onset recurrent oral papillomatosis, new studies are necessary to solve this dilemma [112,113].

Given the increased regression rates of CIN1 and CIN2, particularly in young women, and the higher risk for obstetrical complications after treatment, most guidelines have shifted toward a more conservative approach for young women [74,114]. Nevertheless, the see-and-treat approach has also gained popularity over the years, potentially leading to higher rates of overtreatment [115]. Additionally, hrHPV-based screening has resulted in increased referral rates and more women undergoing biopsy or treatment, thereby elevating the risk for adverse pregnancy outcomes [96].

In this way, it is imperative to optimize the management of CIN in women of reproductive age. Women with potential plans for future pregnancy should receive proper counseling regarding the risks and benefits of both biopsy and excisional treatment. Furthermore, once women with this heightened risk of preterm birth become pregnant, they may benefit from closer surveillance during pregnancy to enhance perinatal outcomes. Further studies are warranted to delve deeper into the role of excision of cervical tissue combined with the presence of cervical disease in the risk of preterm birth [96].

## 6. Safety of HPV Vaccine During Pregnancy

Although the National Advisory Committee on Immunization advises against using the HPV vaccine in pregnant women, accidental administration during pregnancy can happen, particularly because the vaccine is recommended for women of childbearing age. To assess pregnancy outcomes in such cases, manufacturers of both vaccines (the quadrivalent vaccine providing protection against HPV types 6, 11, 16, and 18, and the bivalent vaccine providing protection against HPV types 16 and 18) have established post-marketing pregnancy registries that track exposures occurring within 1 month before the last menstrual period or at any point during pregnancy [116].

Based on data from clinical trials and registries, administering the HPV vaccine during pregnancy has not been linked to negative pregnancy outcomes, such as major malformations, increased risk of stillbirth, preterm birth, small-for-gestational-age infants, or ectopic pregnancy. However, the possibility of an increased risk of spontaneous abortion cannot be entirely ruled out, and the evidence for a causal relationship between HPV vaccination and adverse pregnancy outcomes remains insufficient. Further research should explore the connection between HPV vaccination during these periods and adverse pregnancy outcomes [117].

It remains important to report and monitor any accidental vaccine administrations during or just before pregnancy. If the vaccine is unintentionally given during pregnancy, there is no need for undue concern or consideration of pregnancy termination; however, it is advisable to delay any remaining doses until after the pregnancy is completed. For women who have received the HPV vaccine and plan to become pregnant, there is no need to postpone pregnancy, as the HPV vaccines are inactive. However, it is important to note that the true safety of the HPV vaccine during pregnancy has yet to be definitively confirmed through randomized controlled trials [118].

Thus, given the current state of research and the vaccine’s immunogenicity, women of childbearing age should exercise caution when considering HPV vaccination during these times. Additionally, physicians should be mindful of a woman’s pregnancy status before administering the vaccine and enhance data collection on pregnancy outcomes for future research [116].

## 7. Conclusions

While pregnancy represents a protective and positive factor for the regression of precancerous cervical lesions, the presence of cervical intraepithelial neoplasia, especially of the high-grade type, negatively impacts the evolution and outcome of pregnancy because of proven increased rates of premature delivery and perinatal death.

New complex research is needed to deepen the study of the effects of estrogens and progesterone during pregnancy on the evolution of HPV infection, in order to better understand the pathogenic mechanisms which have an impact on the therapeutic and prognostic management of patients with this pregnancy-associated pathology.
